# Thermoplastic Reaction Injection Pultrusion for Continuous Glass Fiber-Reinforced Polyamide-6 Composites

**DOI:** 10.3390/ma12030463

**Published:** 2019-02-02

**Authors:** Ke Chen, Mingyin Jia, Hua Sun, Ping Xue

**Affiliations:** Beijing University of Chemical Technology, Institute of Plastic Machinery and Engineering, Beijing 100029, China; chenke0903@outlook.com (K.C.); jiamy@mail.buct.edu.cn (M.J.); reignlan@163.com (H.S.)

**Keywords:** glass fiber, thermoplastic composites, pultrusion, thermal analysis, mechanical properties

## Abstract

In this paper, glass fiber-reinforced polyamide-6 (PA-6) composites with up to 70 wt% fiber contents were successfully manufactured using a pultrusion process, utilizing the anionic polymerization of caprolactam (a monomer of PA-6). A novel thermoplastic reaction injection pultrusion test line was developed with a specifically designed injection chamber to achieve complete impregnation of fiber bundles and high speed pultrusion. Process parameters like temperature of injection chamber, temperature of pultrusion die, and pultrusion speed were studied and optimized. The effects of die temperature on the crystallinity, melting point, and mechanical properties of the pultruded composites were also evaluated. The pultruded composites exhibited the highest flexural strength and flexural modulus, reaching 1061 MPa and 38,384 MPa, respectively. Then, effects of fiber contents on the density, heat distortion temperature, and mechanical properties of the composites were analyzed. The scanning electron microscope analysis showed the great interfacial adhesion between fibers and matrix at 180 °C, which greatly improved the mechanical properties of the composites. The thermoplastic reaction injection pultrusion in this paper provided an alternative for the preparation of thermoplastic composites with high fiber content.

## 1. Introduction

In recent years, environmental degradation and the energy crisis have made people aware of the importance of using recyclable materials. Fiber-reinforced thermoplastic composites have attracted more and more attention in various fields, particularly in the automotive industry, due to their manufacturing flexibility and recyclability [1,2]. Long fiber-reinforced thermoplastic composites have been applied to seatback frames and battery storage for hybrid vehicles [3]. As for structural applications requiring improved physical properties, continuous fiber reinforcements are necessary. Thermoplastic composites with continuous fiber have much higher mechanical performance than composites with short or long fiber reinforcements.

Pultrusion is one of the most cost-effective and energy-efficient methods of manufacturing continuous fiber-reinforced composite profiles. Over the past 70 years, thermosetting resins have dominated the pultrusion industry owing to their rapid curing and effective impregnation with low viscosity. However, thermosetting resins have several disadvantages. They are brittle, sensitive to impact, and cannot be recycled. In addition, volatile compounds would be released during the pultrusion process of many thermosetting resins, which is contrary to environmental protection policies. Thermoplastic polymers completely avoid the above shortcomings and offer improved impact strength, damage tolerance, toughness, and reparability [4]. Nevertheless, a relative high viscosity (100–10,000 Pa·s) of molten thermoplastics [5] limits the impregnation ability, resulting in the poor quality of the pultruded products.

Several impregnation techniques for thermoplastic pultrusion have been developed by researchers. One way was the direct melting with high-fluidity polymers, such as high fluidity polyamide-66 (PA-66) [6]. The other way was to put the polymer and fibers in intimate contact prior to the final molding step. Pre-impregnated materials, such as prepreg tapes, commingled fibers, and powder coated towpregs had been well-developed for pultrusion processes [7,8,9,10]. However, there was still a large gap in achieving good impregnation with high fiber content between thermoplastic polymers and thermosetting resins.

In order to solve this problem, reactive processing of thermoplastic composites with anionic ring-opening polymerization of polyamide-6 (PA-6) has been developed by researchers [11,12]. The impregnation of high content fibers can be easily achieved through caprolactam (monomer of PA-6) with extremely low viscosity (5 mPa·s) [11]. It provided an alternative to the manufacture of thermoplastic composites. Gong and Yang prepared all-polyamide composites using resin transfer molding (RTM) in which PA-6 matrix was formed by in-mold anionic polymerization of caprolactam in the presence with PA-66 plain cloth [13]. By comparing the mechanical properties of all-polyamide composites prepared at different temperatures, an optimal mold temperature of 180 °C was found. Yan et al. produced continuous glass fiber-reinforced anionic PA-6 composites through vacuum assisted resin infusion (VARI) [14]. The tensile strength and flexural strength of plain-woven fabric composites reached 434 MPa and 407 MPa, respectively. The anionic ring-opening polymerization of PA-6 can be completed in tens of seconds to 60 min, depending on the different activator–initiator combinations [15]. This provided the possibility for its application in pultrusion. Luisier et al. developed a pilot reactive injection pultrusion line in the base of anionic polymerization of polyamide-12 (PA-12) [16]. Epple et al. successfully prepared thermoplastic composites with anionic PA-6 using a pultrusion process, but the optimization of process condition, properties, and microstructure of pultruded composites were not reported in detail [17].

In this study, a novel thermoplastic reaction injection pultrusion test line was developed to manufacture continuous glass fiber-reinforced PA-6 composites. Using the specifically designed injection chamber, thermoplastic composites with fiber contents up to 70 wt% can be successfully prepared. The effects of temperature of injection chamber on the wet-out of fibers was investigated. Thermal properties and mechanical properties of the composites were also studied under different heating zone temperatures and different fiber contents. Besides, scanning electron microscopy was applied to analyze the microstructure of composite materials.

## 2. Experimental

### 2.1. Materials

**Figure 1:**
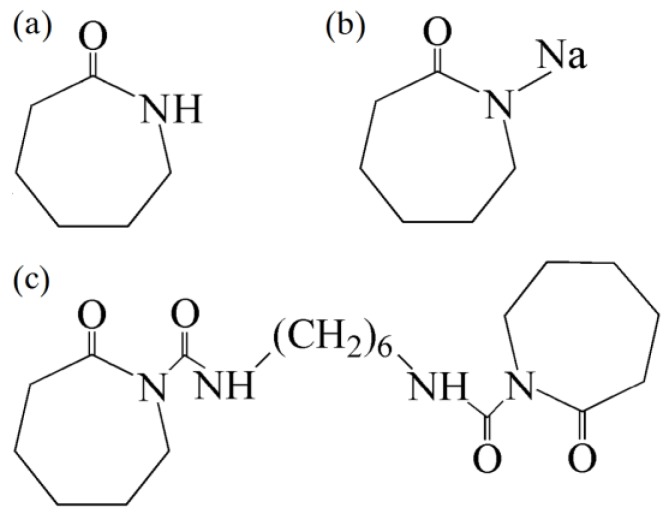
Structures of materials used in this study: (**a**) caprolactam, (**b**) sodium caprolactamate, and (**c**) hexamethylene-1,6-dicarbamoylcaprolactam.

Commercial grade caprolactam (CPL, melting point was 69 °C) was purchased from BASF Co, Heilbronn, Germany. The sodium hydroxide (≥96% by mass, “NaOH”) was kindly provided by Sinopharm Chemical Reagent Co., Ltd, Shanghai, China. Difunctional hexamethylene-1, 6-dicarbamoylcaprolactam (2 mol/kg concentration in caprolactam, ‘C20’) was obtained from Brüggemann Chemical, Heilbronn, Germany, which was used as the activator. The initiator sodium caprolactamate (2 mol/kg concentration in caprolactam, “C10”) was self-prepared via the reaction of caprolactam and NaOH under a vacuum environment. The chemical structures of these materials are presented in Figure 1. C20 concentration of 1.0 mol% and C10 concentration of 2.0 mol% in caprolactam were chosen (C20 is a difunctional activator). Based on the preliminary study [18], these concentrations were intended to shorten the polymerization time while ensuring the molecular weight of PA-6 matrix.

The glass fiber reinforcements were provided by CPIC Co., Ltd, Chongqing, China. These unidirectional E-glass rovings (ECT 4301R) were 2400 tex and treated with a silane coupling agent suitable for PA-6.

**Figure 2:**
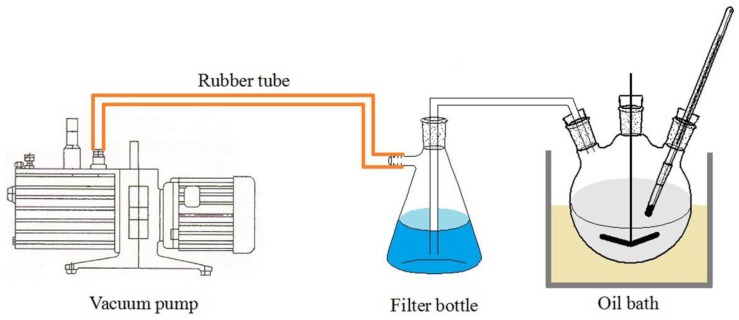
The schematic of the preparation of the initiator C10.

The initiator C10 was particularly sensitive to moisture in the atmosphere and could not remain reactive for long periods of time. Therefore, it had to be prepared before use to ensure reactivity. The schematic of the preparation of the initiator C10 is shown in Figure 2. A three-neck flask containing caprolactam and a certain proportion of NaOH was placed in the oil bath, which was maintained at 135 °C. The mixture was heated to boiling in a vacuum environment for 10–20 min until no particles were present in the three-neck flask, and then the initiator C10 was prepared. The prepared liquid initiator was poured into a 500 mL beaker and cooled to solidify for later use. Nitrogen gas was blown into the beaker to isolate the air before being covered with a glass lid.

### 2.2. Development of Thermoplastic Reaction Injection Pultrusion Test Line

#### 2.2.1. Pultrusion Test Line

**Figure 3:**
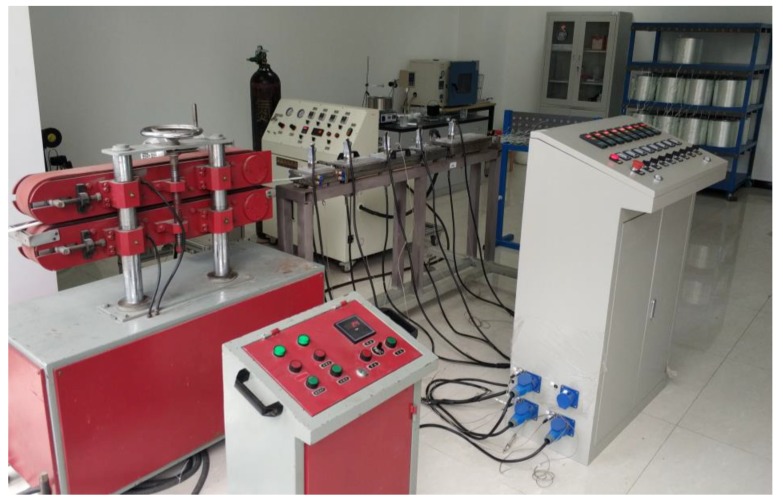
The developed thermoplastic reaction injection pultrusion test line.

The thermoplastic reaction injection pultrusion test line comprises a guiding device for glass fibers, a fiber preheating and arranging unit, an injection unit, a pultrusion die, and a puller, as shown in Figure 3.

**Figure 4:**
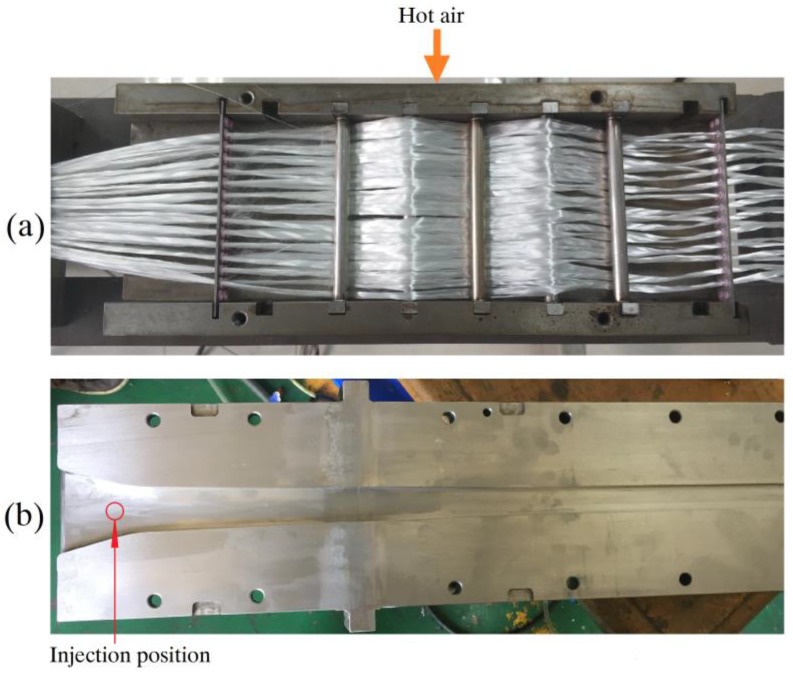
Components of the pultrusion line: (**a**) fibers preheating and arranging unit, and (**b**) the upper part of the pultrusion die.

The glass fibers were preheated and arranged prior to entering the pultrusion die to avoid inhibition of polymerization by moisture on the cold fibers. The fibers were heated by the hot air at 150 °C and heat conduction from the metal tension rollers, which is illustrated in Figure 4a. Two heating techniques worked together to ensure the removal of moisture from the surface of the glass fibers. Tension rollers and guide plates were utilized to make the fiber bundles tightly aligned.

The pultrusion die consisted of three parts: an injection chamber, a heating zone, and a cooling zone. In this study, rectangular profiles with a cross-section of 20 mm × 4 mm were pultruded. A trumpet-shaped injection chamber with a downward angle of 8° was designed. The upper part of the pultrusion die is shown in Figure 4b. This shape of the injection chamber can effectively reduce the backflow of resin and contribute to the impregnation. At the same time, the inclination of 8° ensured that the traction resistance was not that large. It was proved to be feasible in the following experiments. The total length of the pultrusion was determined to be 1.0 m.

#### 2.2.2. Mixing and Injecting of Reaction Materials

**Figure 5:**
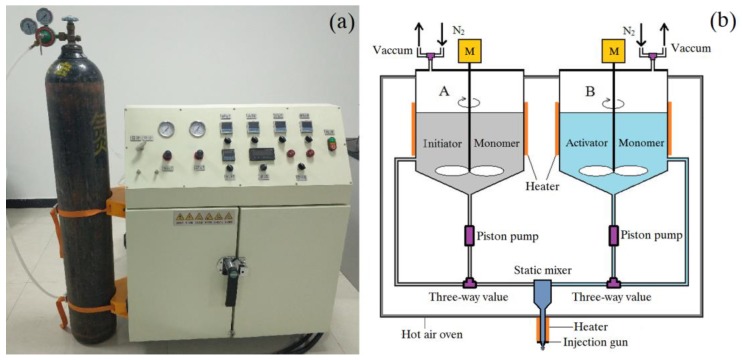
The specially designed resin injection molding (RIM) device: (**a**) overview of RIM device, and (**b**) schematic diagram of the device.

The injection device designed for the anionic polymerization of PA-6 was different from traditional resin injection molding (RIM) device. Specific equipment was required to remove moisture under a high vacuum and high temperature and to protect the molten reaction materials from air. The device had to include the following features: (a) to vacuum the two kinds of liquid reaction materials to drop the moisture content below 300 ppm, (b) to store the molten caprolactam above the melting temperature (69 °C) and protect it from moisture and oxygen, (c) to mix the two components in an accurate ratio, and (d) to inject the both reaction materials into the pultrusion die under certain pressure. These features are achieved by the device shown in Figure 5.

Two kinds of liquid reaction materials were used: the caprolactam and the initiator C10 were in tank A while the caprolactam and the activator C20 were in tank B. When the device was activated, the two reaction materials in the tanks were heated to 135 °C in a vacuum environment. The temperatures of reaction materials could be detected by the thermocouples set on the inner wall of the tanks. After degassing for 20 min, nitrogen was introduced into the tanks and the reaction materials were cooled to an appropriate injection temperature by stirring. Then the two kinds of reaction materials were fed to the static mixer by the pneumatic piston pumps according to a certain ratio (1:1 in this study). The pressure-limiting valves were used to adjust the pressure of the piston pumps to meet the flow requirements. The injection pressure was set to 0.2–0.5 bar to ensure the impregnation based on pultrusion speeds. All the pipes, tanks, and the static mixer were placed in a hot air oven at 100 °C to avoid solidification of caprolactam in advance. The injection gun was also heated with an electric heater to maintain the temperature at 100 °C.

### 2.3. Characterizations

#### 2.3.1. Differential Scanning Calorimetry

Differential scanning calorimetry (DSC) (Mettler Toledo TGA/DSC1, Zurich, Switzerland) analysis was performed to determine the degree of crystallinity (*X*_c_) and melting point (*T*_m_) of the PA-6 matrix of the pultruded composites. The melting enthalpy (*H*_m_) of PA-6 matrix was tested under a nitrogen atmosphere, over a temperature range from 25 °C to 250 °C with a heating rate of 10 °C/min. The PA-6 matrix content (*m*_resin_) was determined using thermogravimetric analysis. Then, the melting enthalpy per unit of mass (∆*H*_m_ = *H*_m_/*m*_resin_) was obtained. *X*_c_ was calculated according to the following equation:

(1) $$X_{c}=\frac{\Delta H_{m}}{\Delta H_{100}}\cdot 100\%$$

where ∆*H*_100_ is the melting enthalpy of fully crystalline PA-6: ∆*H*_100_ = 190 J/g [19].

#### 2.3.2. Thermogravimetric Analysis

Thermogravimetric analysis (TGA) tests of the composites were carried out under a nitrogen flow of 50 mL/min on a TGA instrument (Mettler Toledo TGA/DSC1, Zurich, Switzerland). The temperature range was from 25 °C to 600 °C and the heating rate was 10 °C/min.

#### 2.3.3. Heat Deflection Temperature

The heat deflection temperature (HDT) of the composites was measured using a Thermal Deformation Vicat Softening Point Temperature Tester (KXRW-300CL-3, Taiding test, Chengde, China), following ISO 75-2-2013 [20]. The size of the rectangular specimen was 80 mm × 10 mm × 4 mm. The applied flexural stress was 1.80 MPa and the heating rate was 2 °C/min. HDT was measured at a standard deflection of 0.34 mm.

#### 2.3.4. Mechanical Properties

The mechanical properties of composites were measured by using a universal testing machine (KXWW-20C, Taiding test, Chengde, China). Flexural strength and flexural modulus were tested according to ASTM D-790 [21]. The size of the rectangular specimen was 80 mm × 10 mm × 4 mm and the span was 64 mm. The short beam shear tests were conducted in accordance with ASTM D-2344 [22] and the size of the rectangular specimen was 24 mm × 8.0 mm × 4.0 mm. A three-point bending jig equipped with a 3 mm diameter supports and a 6 mm diameter loading nose was adjusted to a span of 16 mm. The interlaminar shear strength (ILSS) can be calculated according to the following equation:

(2) $$\mathrm{ILSS}=0.75\cdot \frac{F_{m}}{w\cdot t}$$

in which *w* and *t* are the width and thickness of the test specimen. At least five specimens per set of conditions were tested. The densities of the samples were also measured according to ASTM D-792 [23].

#### 2.3.5. Scanning Electron Morphology

Scanning electron morphology (SEM) study was carried out using an electron microscope (S-4700, Hitachi, Tokyo, Japan). Cross-sections of the samples were observed after polishing with water abrasive papers. The morphology of the fracture surface was observed after fracturing the sample in the liquid nitrogen.

## 3. Results and Discussion

### 3.1. Process Feasibility of Thermoplastic Reaction Injection Pultrusion

Different from traditional thermoplastic pultrusion with a melting process, for reaction injection pultrusion, fiber impregnation can be easily achieved by the reaction materials with an ultra-low viscosity. Anionic polymerization occurred in the pultrusion die to manufacture composite materials. This technique was more similar to pultrusion with thermosetting resins, but it required more critical conditions.

The anionic polymerization of caprolactam was particularly sensitive to moisture in the atmosphere. When the relative humidity was higher than 40% in the air, the polymerization was difficult to proceed smoothly. Therefore, an impregnation technique with open resin bath was not ideal. In this study, an injection chamber directly connected to the heating zone was adopted to isolate the air. It avoided the problem of insufficient adhesion of the reaction materials to the fibers when using the open resin bath. The reaction materials injected into the impregnation chamber would move along with the fibers, so a long pot life of reaction materials was also unnecessary. Because commercial caprolactam was used in this study, a strict vacuum dehydration process must be implemented to ensure that the water content of the reaction materials dropped below 300 ppm. The reaction materials in both tanks were heated to boil under 0.01 MPa for 20 min. Before the injection process, the reaction materials should be cooled to 100 °C by stirring to avoid polymerization during the mixing phase.

Another characteristic that must be followed was the high reactivity of reaction materials. Researchers have demonstrated that the combination of initiator C10 and activator C20 provided a very short induction time for polymerization, and the reaction time can be controlled within 2 min [11,24]. In this study, higher contents of activator and initiator were used to accelerate the reaction. Besides, the addition of fibers resulted in higher thermal conductivity of composites than that of neat polymer, resulting in a faster reaction. A pultrusion speed up to 80 cm/min can be achieved. 

### 3.2. Effect of Temperature of Injection Chamber on the Wet-Out of Fibers

When the reaction materials were injected into the injection chamber, the impregnation of the unidirectional glass fiber reinforcements was also carried out simultaneously. The injection chamber needed to be maintained at a suitable temperature to prevent solidification of the caprolactam and to ensure a low viscosity of the reaction materials to impregnate the fibers. Although the reaction temperature range recorded in the literature was 130–170 °C [15], this did not mean that the reaction was not underway below this temperature range. The reaction also took place, but the reaction rate was relatively slow, which can lead to the increase of viscosity of the reaction materials. Both temperature (*T*) and monomer conversion (*α*) had effects on the viscosity of reaction materials (*μ*). This can be described using the following equation [25]:

(3) $$\mu (T,\alpha (T,t))=2.7\times 10^{-7}\cdot \exp(\frac{3525}{T}+17.5\cdot \alpha )$$

**Figure 6:**
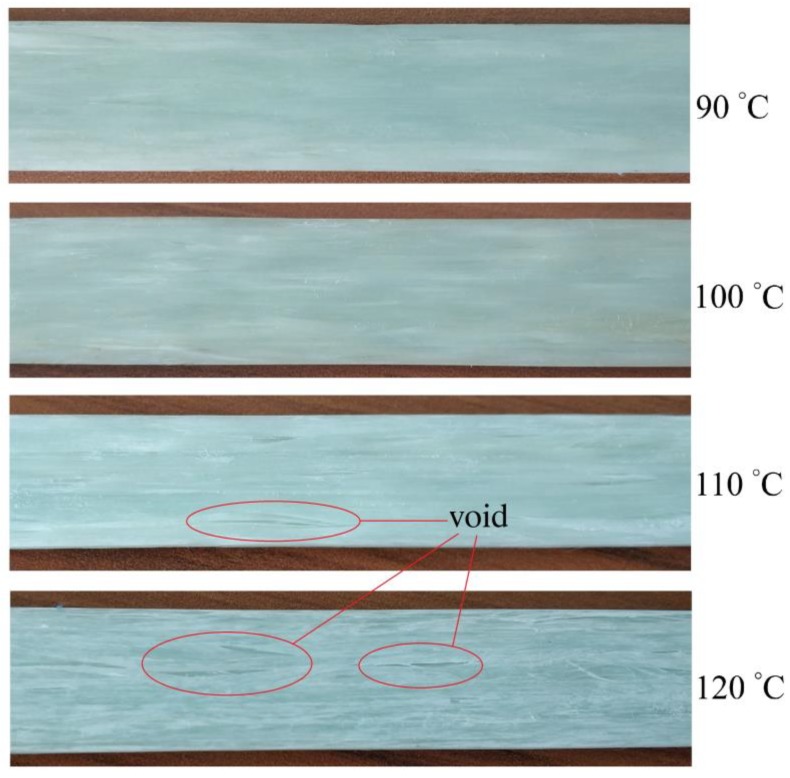
Surfaces of pultruded composites with 70 wt% glass fibers at various temperatures of the injection chamber.

In general, the viscosity should be less than 1 Pa·s to allow the resin to penetrate the fiber reinforcement thoroughly [16]. Therefore, the temperature of the injection chamber was very important and had a great influence on the rheological behavior of the reaction materials, which determined the wet-out of fibers. Surfaces of pultruded composites with 70 wt% glass fibers at various temperatures of the injection chamber are present in Figure 6. It can be seen that some voids appeared on the surfaces of the composites. The higher the temperature, the more serious the phenomenon of voids on the surface of the samples. The surfaces of samples with injection chamber temperatures of 110 °C and 120 °C were unacceptable for qualified pultruded products. The voids on the surface would have a negative effect on the final mechanical properties of the pultruded products.

This was caused by the following effects. Because the injection chamber was directly connected with the heating zone, which had a relative high temperature (150–180 °C), the temperature at the interface between the injection chamber and heating zone was actually higher than the set temperature due to the heat conduction. The reaction materials on the surface of the composites in contact with the heating mold was reacted ahead of time, resulting in a sharp rise in viscosity. Adequate penetration of fiber bundles on surfaces of composites could not be achieved by reaction materials with higher viscosity. This phenomenon was particularly noticeable on the sample surfaces of 110 °C and 120 °C. Samples at 90 °C and 100 °C had flat surfaces because the reaction materials were not heated to the temperature required for the sharp rise in viscosity.

**Figure 7:**
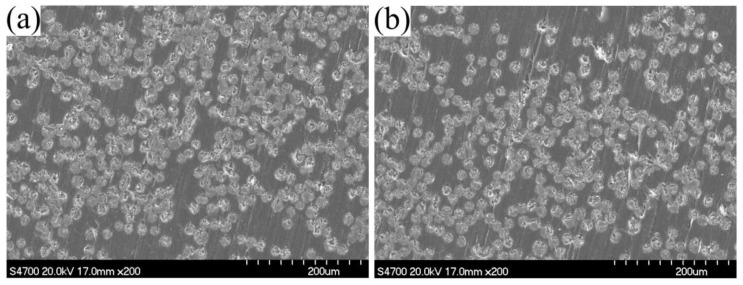
SEM micrographs of the polished composites with injection chamber at 90 °C (**a**) and 100 °C (**b**).

SEM micrographs of the polished composites with the injection chamber at 90 °C and 100 °C are shown in Figure 7. In spite of scratches caused by polishing, the pictures confirmed excellent impregnation. The void content of the composite samples was nearly zero. Therefore, it was a good choice to set the temperature of the injection chamber at 90–100 °C. For the following sections, the temperature of the impregnation chamber was set at 100 °C in order to get a faster reaction speed.

### 3.3. Effect of Heating Zone Temperature on the Thermal Properties of PA-6 Matrix of the Pultruded Composites

In general, the molding die for pultrusion included a high temperature zone for the resin reaction and a low temperature zone for cooling and shaping. In this study, the temperature of the cooling zone was set to be only 20 °C lower than that of the heating zone. This was because the PA-6 matrix of the composites was a semi-crystalline polymer and a slow cooling rate can improve the crystallinity, which had a great influence on the mechanical properties of the polymer. Due to the addition of fibers, the heat resistance of composites was greatly improved and the feasibility of the cooling zone with a relative high temperature was ensured. The specific process temperatures and pultrusion speed are shown in Table 1. High die temperature can accelerate the reaction, thus achieving a high pultrusion speed.

**Figure 8:**
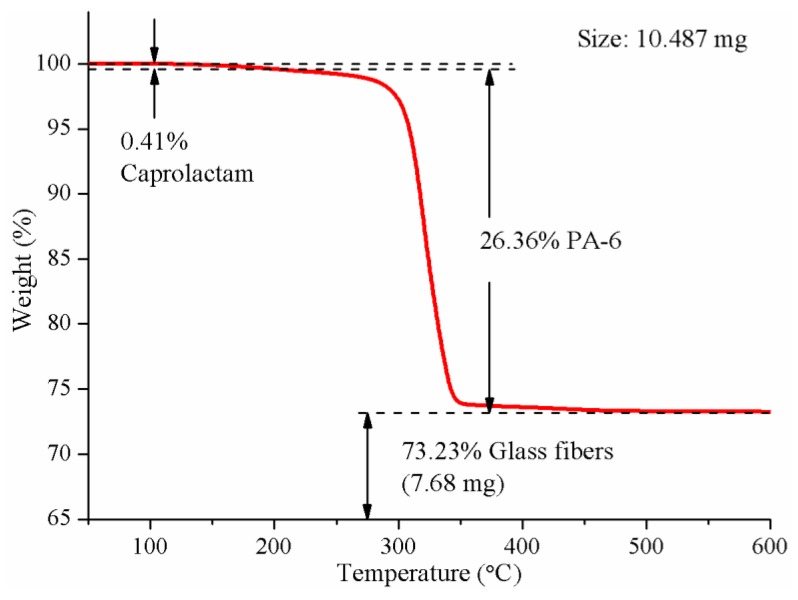
A typical TGA diagram of the composites pultruded with heating zone temperature of 180 °C.

In order to determine the crystallinity of PA-6 matrix in the composites, TGA tests should be carried out to determine the proportion of the matrix. Figure 8 is a typical TGA diagram of the composites pultruded with a heating zone temperature of 180 °C. It can be seen from the figure that the PA-6 matrix decomposed completely at 500 °C, leaving only glass fibers. The weight percent of glass fibers was 73.23%. There may also be residues in the composites, which were not involved in the reaction. According to the literature [26], the residual caprolactam was volatilized below 200 °C. Except for that part, the proportion of matrix PA-6 can be determined.

Since the reaction was carried out below the melting point of PA-6, polymerization and crystallization occurred simultaneously. The temperature of the pultrusion die had a great effect on the crystallinity and melting point of PA-6.

**Figure 9:**
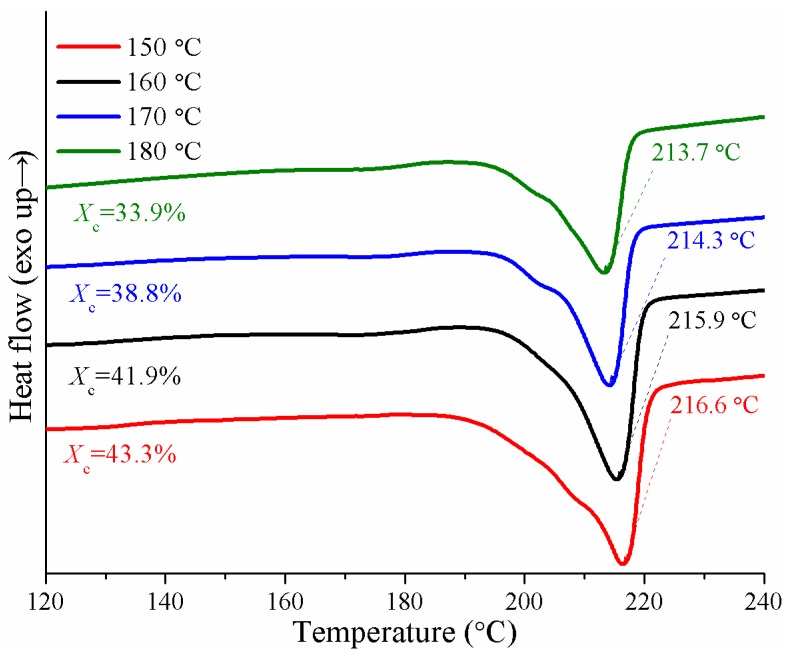
Degree of crystallinity and melting point of PA-6 matrix for various heating zone temperature measured by DSC.

Figure 9 shows that the degree of crystallinity of the PA-6 matrix in composites gradually decreases as the heating zone temperature increased from 150 to 180 °C. The reason was that the higher the polymerization temperature was, the stronger the thermal motion of the polymer chain was, resulting in a lower equilibrium degree of crystallinity, which meant that more time was needed to achieve a crystallization equilibrium [12]. With the increasing die temperature, the increase of molecular weight of the PA-6 matrix caused by the branching reaction was another reason [14]. The increase in molecular weight enhanced the entanglement between polymer chains, which limited the internal rotation of polymer chains, thus affecting the orderly accumulation of molecular chains, and had a negative impact on the crystallization behavior of the PA-6 matrix. The presence of fibers also made the crystallinity of PA-6 matrix in composites higher than that of the pure PA-6 that was reported [12]. Due to the heterogeneous nucleation, fibers may act as nucleating agents in the composites. What is more, in the presence of fiberglass, the total heat release of the resin system was lower, and more polymers had the tendency of crystallization.

The melting point of the PA-6 matrix decreased by more than 3 °C with the increase of temperature from 150 to 180 °C. This may have been due to the transformation of crystal structure caused by a branching reaction [27]. The *α*-structure crystal (*T*_m_ = 220 °C) and *γ*-structure crystal (*T*_m_ = 214 °C) appeared in the anionic PA-6 simultaneously. The branching reaction led to an increase in the content of *γ*-structure crystals, thereby the melting point was reduced.

### 3.4. Effect of Heating Zone Temperature on the Mechanical Properties of the Pultruded Composites

**Figure 10:**
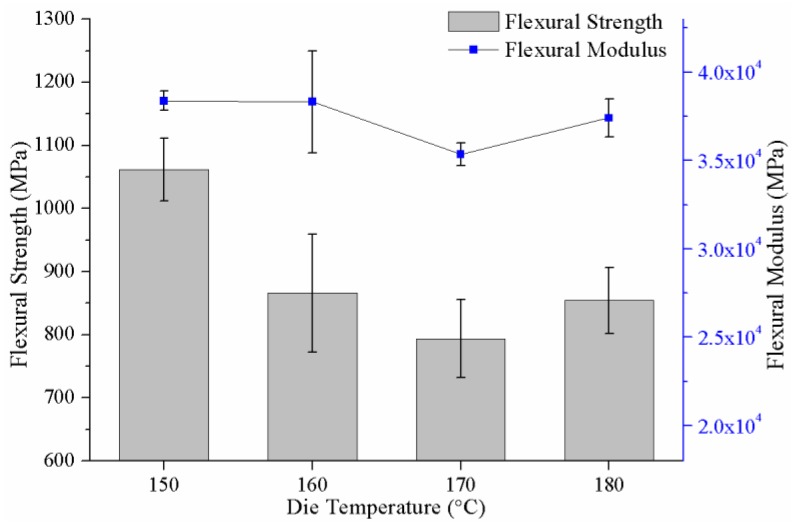
Flexural properties of the pultruded composites depending on the heating zone temperature.

Four composite samples with 70 wt% glass fibers depending on the heating zone temperatures were tested to investigate the flexural properties and interlaminar shear strength (ILSS). From Figure 10, we can see that in the heating zone temperature of 150 °C, the flexural strength and the flexural modulus reached the maximum value of 1061 MPa and 38,384 MPa, respectively. As the heating zone temperature rose from 150 °C to 170 °C, the flexural properties of the composites decreased gradually, which was consistent with the decreasing trend of crystallinity of the PA-6 matrix in Figure 9. When the heating zone temperature was further increased to 180 °C, the flexural properties of the composites were improved. This variation tendency can be explained using the following reasons. In the heating zone temperature range of 150 °C to 170 °C, as with most semi-crystalline polymers, the mechanical properties of the PA-6 matrix of the composites mainly depended on the degree of crystallinity. Therefore, the decrease of crystallinity would reduce the mechanical properties of PA-6 matrix, resulting in a decrease in the flexural properties of the composites. As the temperature reached 180 °C, the interfacial bonding between fibers and PA-6 matrix was greatly improved and played a major role in the flexural properties of the composites, which can be demonstrated by the following analysis of ILSS of the composites.

**Figure 11:**
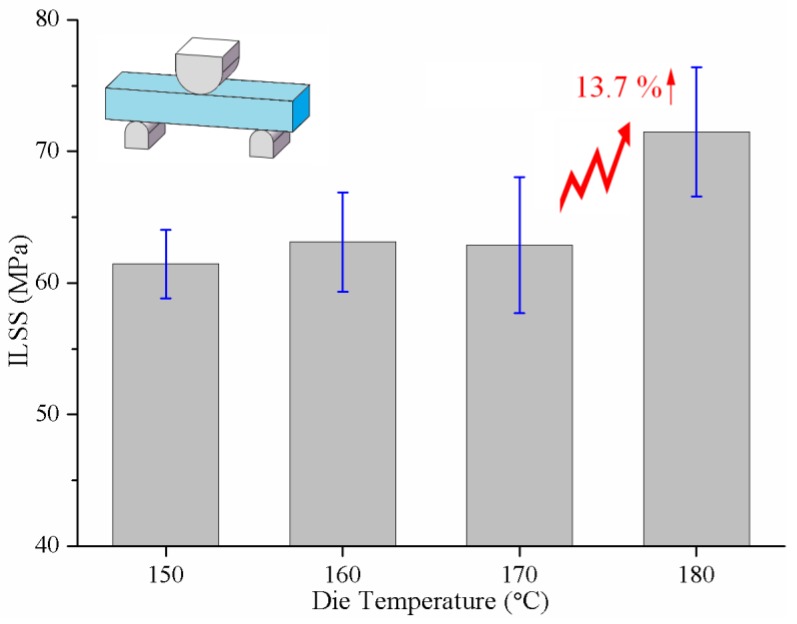
The interlaminar shear strength of the pultruded composites depending on heating zone temperature.

Figure 11 shows the test results of the ILSS of specimens with 70 wt% glass fibers at various heating zone temperatures. When the temperature was in the range of 150 °C to 170 °C, there was little difference between the ILSS of the pultruded composites. However, ILSS of the composites reached 71.5 MPa at heating zone temperature of 180 °C, which was 13.7% higher than that at 170 °C. This was because the de-blocking of the activator C20 occurred to a larger extend at heating zone temperature of 180 °C and more free isocyanate groups were generated, which increased the chance of the bond formation of aminosilane urea links on the glass surface [12,27]. The interfacial bonding between the fibers and PA-6 matrix was greatly enhanced.

It can be concluded that it was the best choice to set 180 °C as the temperature of the heating zone. Not only the pultruded composites exhibited relatively excellent mechanical properties, but a high pultrusion speed could also be achieved.

### 3.5. Effect of Fiber Content on the Properties of the Pultruded Composites

Table 2 illustrates the properties of the pultruded continuous glass fiber-reinforced PA-6 composites with different fiber contents. The density and HDT of pultruded composites increased with increasing fiber content due to the higher specific gravity and thermal stability of the glass fiber. The composite material reached an HDT much higher than that of pure PA-6, reaching about 200 °C. With the increase of fiber content, the flexural strength, flexural modulus, and ILSS of composites also increased. This was because, as the reinforcement, the glass fiber played a dominant role in the mechanical properties of composites.

### 3.6. Scanning Electron Microscopy Analysis of the Pultruded Composites

**Figure 12:**
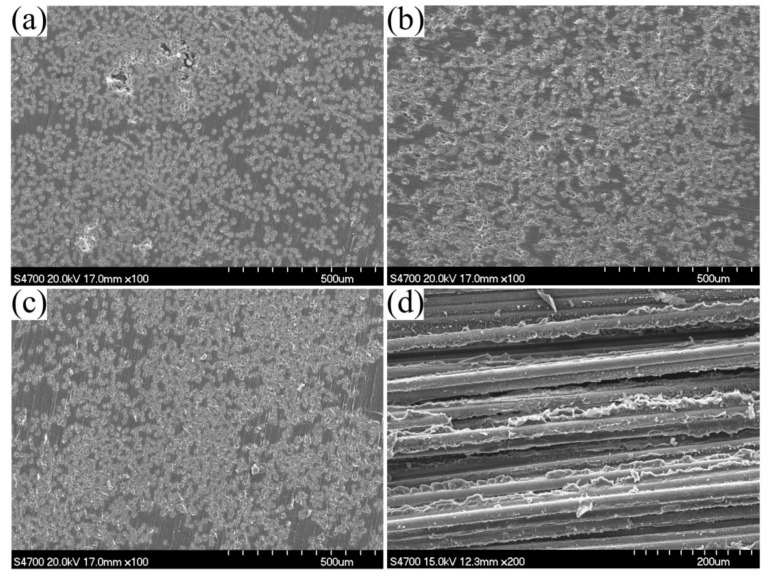
SEM micrographs of the pultruded composites: (**a**) cross sections with 50 wt% glass fibers, (**b**) cross sections with 60 wt% glass fibers, (**c**) cross sections with 70 wt% glass fibers, and (**d**) fracture surfaces with 70 wt% glass fibers, all at a heating zone temperature of 180 °C.

The microstructures of pultruded composites were observed using scanning electron microscopy. The cross sections of the pultruded composites with different fiber contents after polishing are shown in Figure 12a–c. With the increase of fiber content, the fibers became denser and the fibers dispersed more and more evenly. Except for the cross-sections in Figure 12a, there were no indications of specific regions where the fibers bundles form for cross-sections with fiber contents of 60 wt% and 70 wt%. However, micron-sized voids were found in the cross section of 50 wt% products, which may be due to the high resin contents and resin shrinkage. There was almost no void in the cross-sections of the pultruded composites with fiber contents of 60 wt% and 70 wt%. Figure 12d shows the fracture surfaces of the composites with 70 wt% glass fibers prepared at a heating zone temperature of 180 °C. It can be seen from the figure that there was a good interfacial bond between the fiber and the matrix under the action of the coupling agent.

## 4. Conclusions

Continuous glass fiber-reinforced PA-6 composites were successfully pultruded via the anionic ring-opening polymerization of caprolactam. As the injection chamber temperature was set at 90–100 °C, good fiber impregnation and a smooth surface of pultruded composites were achieved. The temperature of the heating zone had significant effects on both the thermal properties of the PA-6 matrix and mechanical properties of composites. When the heating zone temperature was 150 °C, the crystallinity of the PA-6 matrix was the highest (43.3%) and flexural strength and flexural modulus of composites reached the maximum of 1061 MPa and 38,384 MPa, respectively. However, the highest interlaminar shear strength (ILSS) of composites occurred at 180 °C, reaching 71.5 MPa, due to the interfacial bonding between the fibers and PA-6 matrix was greatly improved by de-blocking of the activator C20. Mechanical properties, heat deflection temperatures, and densities of pultruded composites increased with increasing glass fiber content. Micron-sized voids were found in cross-sections of composites with 50 wt% fibers, but not in composites with higher fiber contents. Great interfacial bonding between the fibers and the matrix was also observed using scanning electron microscopy.

## Figures and Tables

**Table 1:** The specific process parameters of the thermoplastic reaction injection pultrusion.

Temperature (°C)			Pultrusion Speed (cm/min)
Injection Chamber	Heating Zone	Cooling Zone	
100	150	130	30
	160	140	40
	170	150	60
	180	160	80

**Table 2:** The properties of pultruded composites with different fiber contents.

Fiber Contents (wt%)	Roving Numbers (2400 tex)	Density (kg/m^3^)	HDT (°C)	Flexural Strength (MPa)	Flexural Modulus (MPa)	ILSS (MPa)
50	26	1.56 × 10^3^	197.8	731.64	24,526.76	51.14
60	34	1.72 × 10^3^	201.6	834.36	33,664.22	58.85
70	42	1.84 × 10^3^	204.5	865.82	36,991.41	63.11
